# Supplementary material for: Antibacterial effects of Kampo products against pneumonia causative bacteria
Source: PLoS One. 2024 Oct 28;19(10):e0312500. doi: 10.1371/journal.pone.0312500 (PMC11515972; doi:10.1371/journal.pone.0312500)
Supplement: S1 Table — (PPTX) [file pone.0312500.s001.pptx]

## Slide 1
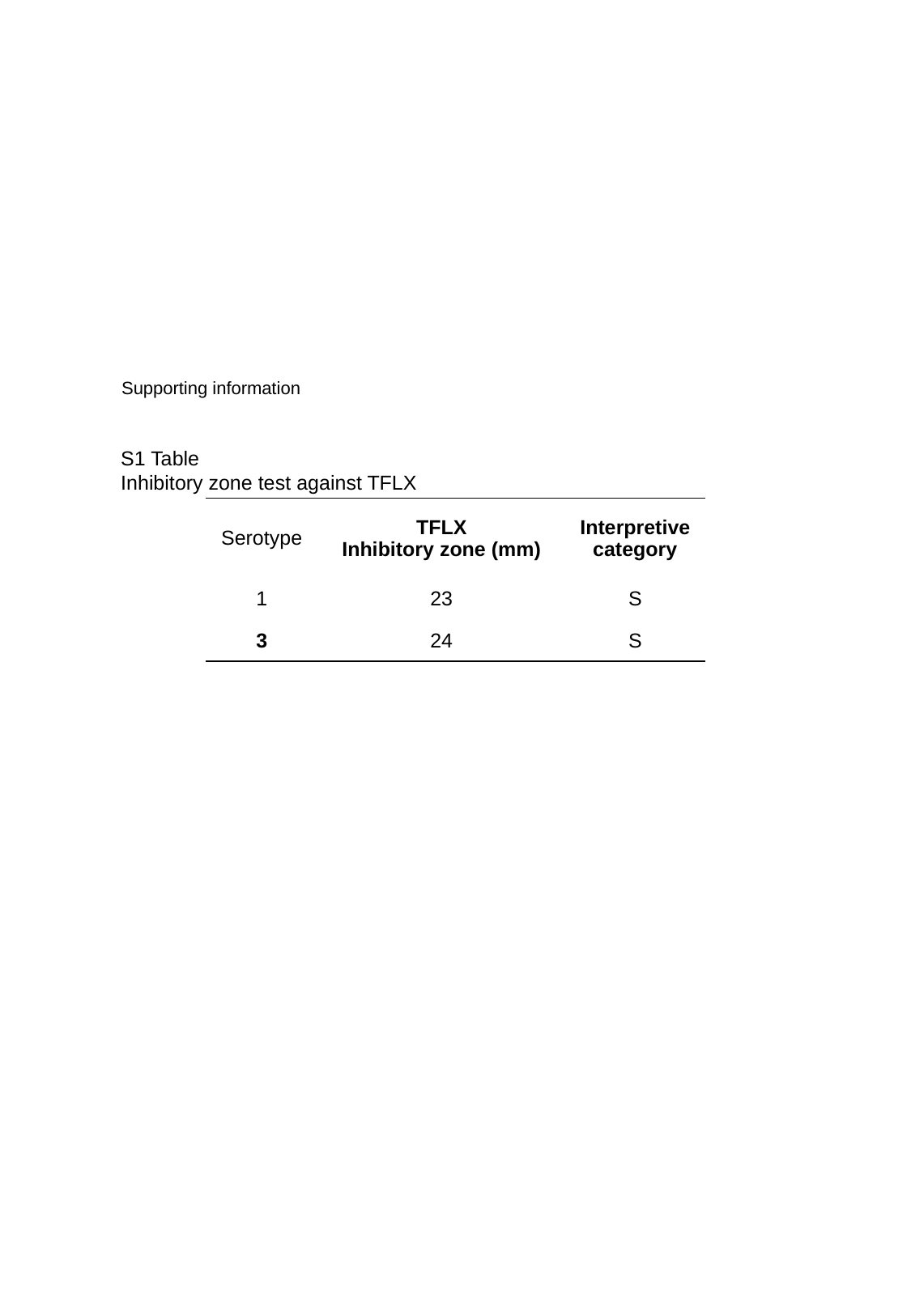

Supporting information
S1 Table
Inhibitory zone test against TFLX
| Serotype | TFLX Inhibitory zone (mm) | Interpretive category |
| --- | --- | --- |
| 1 | 23 | S |
| 3 | 24 | S |
