# Supplementary material for: Antibacterial effects of Kampo products against pneumonia causative bacteria
Source: PLoS One. 2024 Oct 28;19(10):e0312500. doi: 10.1371/journal.pone.0312500 (PMC11515972; doi:10.1371/journal.pone.0312500)
Supplement: S2 Table — (PPTX) [file pone.0312500.s002.pptx]

## Slide 1
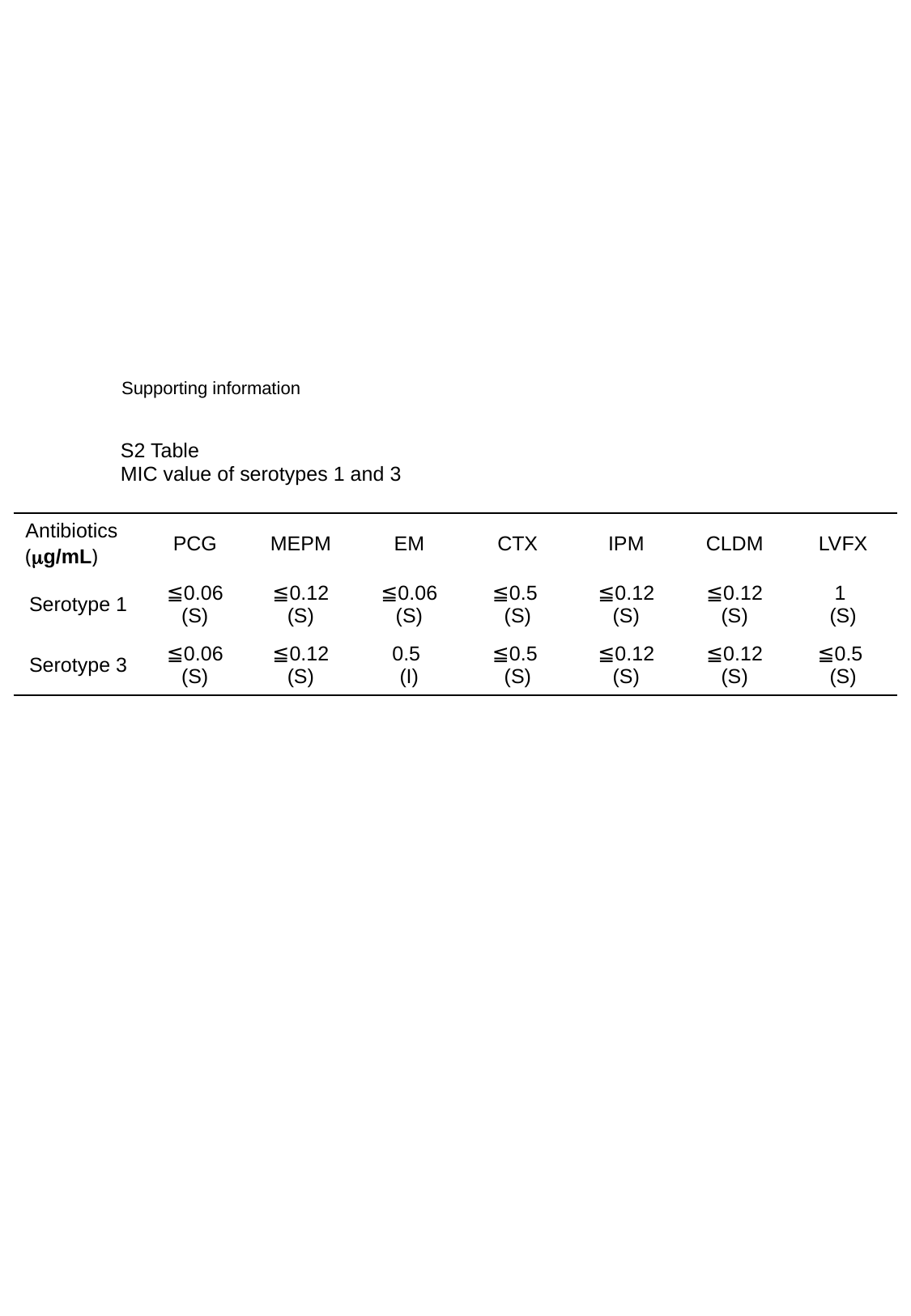

Supporting information
S2 Table
MIC value of serotypes 1 and 3
| Antibiotics (mg/mL) | PCG | MEPM | EM | CTX | IPM | CLDM | LVFX |
| --- | --- | --- | --- | --- | --- | --- | --- |
| Serotype 1 | ≦0.06 (S) | ≦0.12 (S) | ≦0.06 (S) | ≦0.5 (S) | ≦0.12 (S) | ≦0.12 (S) | 1 (S) |
| Serotype 3 | ≦0.06 (S) | ≦0.12 (S) | 0.5 (I) | ≦0.5 (S) | ≦0.12 (S) | ≦0.12 (S) | ≦0.5 (S) |
