# Supplementary material for: Antibacterial effects of Kampo products against pneumonia causative bacteria
Source: PLoS One. 2024 Oct 28;19(10):e0312500. doi: 10.1371/journal.pone.0312500 (PMC11515972; doi:10.1371/journal.pone.0312500)
Supplement: S2 Dataset — (PPTX) [file pone.0312500.s005.pptx]

## Slide 1
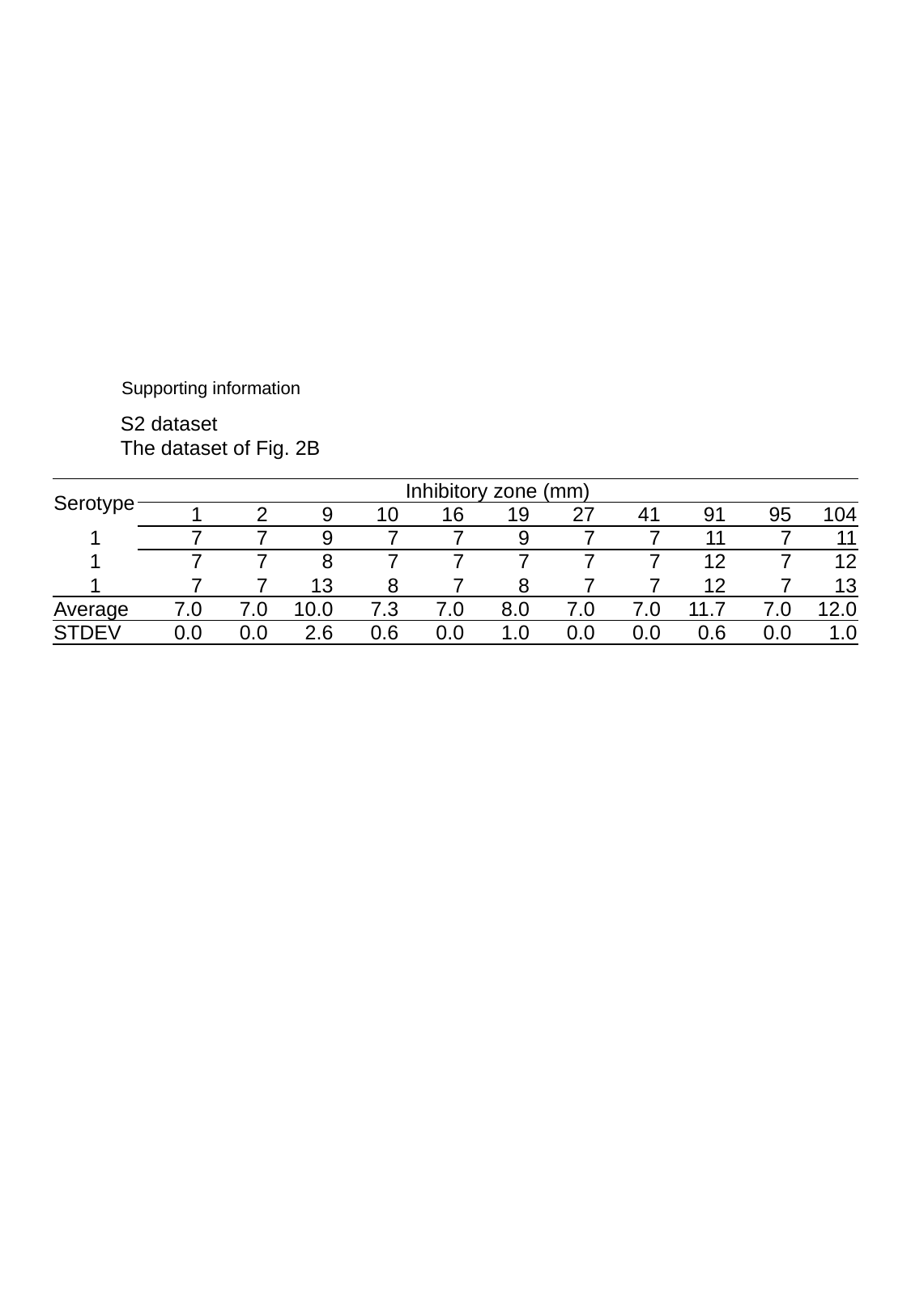

Supporting information
S2 dataset
The dataset of Fig. 2B
| Serotype | Inhibitory zone (mm) | | | | | | | | | | |
| --- | --- | --- | --- | --- | --- | --- | --- | --- | --- | --- | --- |
| | 1 | 2 | 9 | 10 | 16 | 19 | 27 | 41 | 91 | 95 | 104 |
| 1 | 7 | 7 | 9 | 7 | 7 | 9 | 7 | 7 | 11 | 7 | 11 |
| 1 | 7 | 7 | 8 | 7 | 7 | 7 | 7 | 7 | 12 | 7 | 12 |
| 1 | 7 | 7 | 13 | 8 | 7 | 8 | 7 | 7 | 12 | 7 | 13 |
| Average | 7.0 | 7.0 | 10.0 | 7.3 | 7.0 | 8.0 | 7.0 | 7.0 | 11.7 | 7.0 | 12.0 |
| STDEV | 0.0 | 0.0 | 2.6 | 0.6 | 0.0 | 1.0 | 0.0 | 0.0 | 0.6 | 0.0 | 1.0 |
