# Supplementary material for: Antibacterial effects of Kampo products against pneumonia causative bacteria
Source: PLoS One. 2024 Oct 28;19(10):e0312500. doi: 10.1371/journal.pone.0312500 (PMC11515972; doi:10.1371/journal.pone.0312500)
Supplement: S3 Dataset — (PPTX) [file pone.0312500.s006.pptx]

## Slide 1
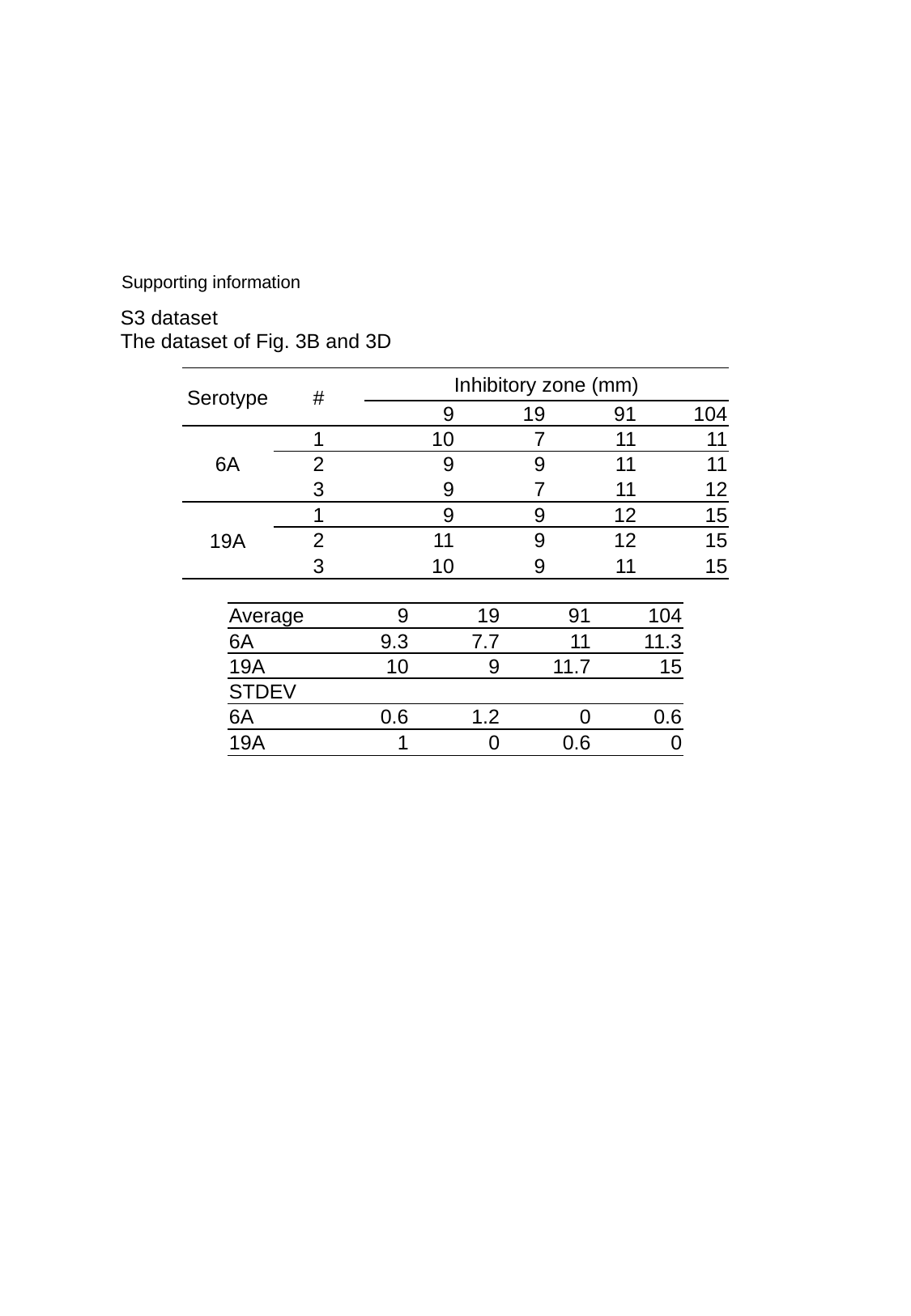

Supporting information
S3 dataset
The dataset of Fig. 3B and 3D
| Serotype | # | Inhibitory zone (mm) | | | |
| --- | --- | --- | --- | --- | --- |
| | | 9 | 19 | 91 | 104 |
| 6A | 1 | 10 | 7 | 11 | 11 |
| | 2 | 9 | 9 | 11 | 11 |
| | 3 | 9 | 7 | 11 | 12 |
| 19A | 1 | 9 | 9 | 12 | 15 |
| | 2 | 11 | 9 | 12 | 15 |
| | 3 | 10 | 9 | 11 | 15 |
| Average | 9 | 19 | 91 | 104 |
| --- | --- | --- | --- | --- |
| 6A | 9.3 | 7.7 | 11 | 11.3 |
| 19A | 10 | 9 | 11.7 | 15 |
| STDEV | | | | |
| 6A | 0.6 | 1.2 | 0 | 0.6 |
| 19A | 1 | 0 | 0.6 | 0 |
