# Supplementary material for: Antibacterial effects of Kampo products against pneumonia causative bacteria
Source: PLoS One. 2024 Oct 28;19(10):e0312500. doi: 10.1371/journal.pone.0312500 (PMC11515972; doi:10.1371/journal.pone.0312500)
Supplement: Attachment — Submitted filename: Response to Reviewers.docm [file pone.0312500.s008.docm]

**Reviewers’ comments:**

**Reviewer #1:**

Thank you very much for your time and the precious comments. We describe the responses to the comments below.

1. In this study, they demonstrated that several Kampo products are effective against S. pneumoniae and S. aureus, which are Gram-positive bacteria. However, the Kampo products did not show effectiveness against K. pneumoniae and E. coli, both that are Gram-negative bacteria. I think it is important to discuss that difference in effectiveness of these Kampo products against Gram-negative and Gram-positive bacteria in terms of components, structure or other relevant factors of Kampo products.

**Response:** Thank you for the important comment. According to the reviewer’s suggestion, we examined components and structures from selected *Kampo* products. However, we could not find general structural features of the *Kampo* components. Notably, we discussed why *E. coli* and *K. pneumoniae* were resistant to the *Kampo* products. We added the description in the *Discussion* section (L260-272, p15-16).

2. In diffusion assay, they showed no inhibitory effects of Kampo products including (9) and (104) on K. pneumoniae and E. coli (Figure 4A and B). On the contrary, they provided MIC values of (9) and (104) against the both bacteria by another assay (Table 4). Are the data consistent with each other?

**Response:** The data are consistent. In the test guideline by CLSI, a 0.5 McFarland bacteria suspension is used in both assays. These methods are quite different; in diffusion assay, the bacterial suspension is swabbed to agar plates, and each *Kampo* suspension is dropped into the prepared hole on the agar plate. For evaluation of MIC, the bacterial suspension (50 μL) is applied to serially diluted *Kampo* suspensions in 96-well plates. Therefore, the number of bacteria and the amount of *Kampo* suspensions are not equal between both assays.

Minor points

Title: Kampo medicines products -> Kampo products

LINE161 and 164: [91] -> (91)

Figure 1B: A black bar corresponding to 10 -> A white bar

Figure 2B: Black bars corresponding to 10 and 19 -> White bars

Figure 3B: A black bar corresponding to 19 -> A white bar. Add an explanation in Figure legend.

Figure 4B: A black bar corresponding to 16 -> A white bar

**Response:** We have corrected all the minor points you have pointed out.

**Reviewer #2:**
Thank you very much for your time and the important comments. We describe the responses to the comments below.

Major Assessment
This paper reports the MIC and MBC of Kampo medicines against common causative bacteria in respiratory tract and soft tissue infections, including clinical isolates with notable drug resistance, using CLSI-standardized microdilution methods. The results are presented clearly.
However, as discussed, the inhibitory concentrations for pneumonia treatment need careful consideration regarding pharmacokinetics and clinical relevance. The paper should provide a detailed explanation of the gap between the MIC/MBC values and achievable blood/tissue concentrations. Moreover, the paper lacks discussion on which chemical components or combinations in the Kampo medicine might inhibit bacterial growth, which should be addressed if hypotheses exist.
**Response:** Thank you for the critical comment. In this study, we showed the inhibitory concentrations of *Kampo* products against *S. pneumoniae* and *S. aureus* tended to be relatively high. The level of inhibitory effect is considered unpreferable for treating pneumonia caused by respiratory bacteria. On the other hand, *Kampo* products contain crude drugs, which are known to be effective against some bacteria even at low concentrations (Liao et al., Pharm. Biol., 2013; Fukamachi et al., Evid. Based Complement. Alternat. Med., 2015). We explored crude drugs in Sho-saiko-To (9) and Shin'i-seihai-To (104) and found that the *Kampo* products contained Ou-gon as a common crude drug. Ou-gon has known to have antifungal properties (Da et al., Nat. Prod. Commun., 2016; Da et al., Sci. Rep., 2019). Therefore, it is suggested that Ou-gon might have the potential to exhibit antimicrobial activity against *S. pneumoniae* and *S. aureus* at low concentrations. We added the description in the *Discussion* section (L284-303, p16-17).

Minor Recommendations
L16, p2: The term "biological effect" should be revised to "antimicrobial effect" since the study does not investigate host bioavailability or cellular biology.

**Response:** Thank you for the comment. We replaced the term in the *Abstract* section (L19, p2).

L16, p2: Add a description of how common respiratory pathogens can colonize the upper respiratory tract or oral cavity and invade the lower respiratory tract, causing pneumonia.
**Response:** We added the description in the *Introduction* section (L39-43, p3).

L43, p3: Cite studies indicating the decreasing trend of community-acquired infections by PRSP and quinolone-resistant pneumococci over the past 25 years with one or two appropriate references and discuss whether this trend is likely to continue but concisely.
**Response:** We added the description in the *Introduction* section (L47-54, p3).

Introduction: While it is crucial to note that Kampo medicine exhibits antimicrobial effects, the mechanisms should be distinguished clearly between protozoa, viruses, bacteria, fungi, etc. The physiological non-realistic high concentrations only achievable on mucosal surfaces versus the pharmacokinetically feasible concentrations in the blood and target organs should be explicitly discussed.
**Response:** We have rewritten the text in the *Introduction* section in detail (L66-75, p4). Also, we added the description about the pharmacokinetics of *Kampo* products in the *Discussion* section (L284-303, p16-17).

L76, p5: Provide a brief explanation for using Todd-Hewitt broth for pneumococci versus nutrient-rich media for other bacteria, particularly regarding the susceptibility and growth phase considerations. For example, considering the fragility or unstable viability of pneumococcal live microbes on a simple agar surface.

**Response:** We added the description in the *Materials and Methods* section (L90-91, p6).

Pneumococci MIC and MBC Testing: Were standard strains, such as ATCC, included in the parallel testing, and were MIC and MBC values measured for Serotype 3 pneumococci against antibiotics?

**Response:** We used the clinically isolated strains only in this study. According to your comments, we tested the MIC and MBC values of serotype 1 and serotype 3. The data are added in *Supplementary information* as S1-3 Table.

L93, p5: Clarify the volume of the bacterial suspension and the agar used in the growth inhibitory assay.

**Response:** According to the test guideline by CLSI, we have inoculated the bacteria using a swab. We corrected the description in the *Materials and Methods* section (L107-109, p6-7).

Figure 4B: Ensure the inhibitory effect or lack thereof for Kampo No. 16 is clearly interpreted and explained.

**Response:** *Kampo* No. 16 lacks the inhibitory effect. We have corrected the Figure 4B.

Overall Structure
The inclusion of figure legends within the results section should be reviewed for adherence to academic journal standards and, if necessary, addressed by the editorial team.

**Response:** We have written the manuscript in accordance with the guidelines provided by PLOS ONE.

<https://journals.plos.org/plosone/s/file?id=wjVg/PLOSOne_formatting_sample_main_body.pdf>
