# Supplementary material for: Antibacterial effects of Kampo products against pneumonia causative bacteria
Source: PLoS One. 2024 Oct 28;19(10):e0312500. doi: 10.1371/journal.pone.0312500 (PMC11515972; doi:10.1371/journal.pone.0312500)
Supplement: Attachment — Submitted filename: Response to Reviewers2_Final.docx [file pone.0312500.s009.docx]

**Reviewers’ comments:**

**Reviewer #1:**

Thank you very much for your time to review our article.

**Reviewer #2:**
Thank you very much for your time and the precious comments. We describe the responses to the comments below.

In last addition, I would like to suggest a minor modification regarding the discussion on the limited efficacy of Kampo compounds against Gram-negative bacteria. Specifically, you mentioned the role of LPS (lipopolysaccharides) as a potential barrier. It would strengthen the manuscript if you could incorporate a few more relevant citations and consider the following explanation:

The LPS-rich outer membrane of Gram-negative bacteria is known to act as a barrier against many antimicrobial agents. Scutellaria baicalensis (Ougon) contains flavonoids such as baicalin and baicalein, which have been reported to inhibit bacterial growth, particularly in Gram-positive bacteria, by targeting cell wall synthesis and DNA replication. LPS-containing outer membrane may resist against Ougon incorporation. Additionally, these compounds exhibit various physiological activities within the host. Incorporating a brief discussion of these established effects, perhaps before the ACR family discussion on page 15, could further substantiate the study's relevance.

**Response:** Thank you for your comment. We added the description in the *Discussion* section (L264 p.15, L301-302, L304-312 p.17). Also, we eliminated irrelevant descriptions from the text.

While it is hypothesized that the rise in blood concentrations of these lipophilic flavonoids, such as baicalin and baicalein, occurs relatively rapidly following standard oral dosing, their sufficiency in achieving therapeutic levels in the inflamed lung tissue remains uncertain. Current research on their in vivo antimicrobial activity is still limited, and this should be carefully examined in future studies. If this line of reasoning aligns with your findings, it might be worthwhile to include it in the discussion. However, given that your investigation focuses on the overall effects of the complex mixture within Kampo medicine, where flavonoids are not the sole active components, this addition is not mandatory.
**Response:** Thank you for your comment. The metabolism and blood concentration levels of some flavonoid compounds have been investigated in detail. Also, as per the reviewer’s comment, it is unclear whether the concentrations of flavonoids in inflamed lung tissues are sufficient for therapeutic levels. Furthermore, it is suggested the possibility that Baicalin exhibits side effects such as interstitial pneumonia and liver dysfunction. Therefore, I think that it is necessary to carefully study their *in vivo* activity. However, we did not describe the discussion by considering that the present study focused on the efficacy of *Kampo* products, which are a mixture of *Shouyaku* (crude drug), but not flavonoids, as described by the reviewer. I think that this is beyond the scope of discussion in the present study.
